# Impact of Printing Angle and Layer Height on the Mechanical Strength of PLA Reinforced with Chopped Carbon Fibres Using FDM 3D Printing

**DOI:** 10.3390/polym17223069

**Published:** 2025-11-19

**Authors:** Oscar Araque, Luz Adriana Sánchez-Echeverri, Ivonne X. Cerón

**Affiliations:** 1 Department of Mechanical Engineering, Universidad de Ibagué, Ibagué 730001, Colombia; 2 Departamento de Fisica, Facultad Ciencias, Universidad del Tolima, Barrio Santa Helena A.A. 546, Ibagué 730006299, Colombia; lasancheze@ut.edu.co; 3 Departamento de Producción y Sanidad Vegetal, Facultad Ingeniería Agronómica, Universidad del Tolima, Barrio Santa Helena A.A. 546, Ibagué 730006299, Colombia; ixcerons@ut.edu.co

**Keywords:** PLA carbon (PLA-CF), FDM (Fused Deposition Modelling), tensile failure, printing angle, layer height, ANOVA analysis

## Abstract

This research addresses the inherent limitations of low mechanical strength in FDM-printed materials by studying Carbon Fibre-reinforced Polylactic Acid (PLA-CF) composites. The low strength limitation of PLA-CF in FDM requires identifying the most suitable print angle and layer height parameters. This study maximises its structural robustness, filling a knowledge gap regarding its combined effect on tensile and flexural strength. The main objective was to find the best printing angle and layer height to improve mechanical performance, an important requirement for advancing additive manufacturing applications. A total of 210 FDM-printed specimens of the PLA-CF material were subjected to uniaxial tensile (ASTM D3039) and three-point bending (ASTM D790) tests, systematically varying the printing angles (0–90°) and layer heights of 0.1, 0.2, and 0.3 mm, following a full experimental design matrix. The ANOVA method has been used to determine the significant effect of factors on the established parameters. The findings indicated that both factors had a pronounced effect on the mechanical strength. Printing at lower angles (0° and 15°) provided, on average, greater resistance under tension (up to ~3920 N for a layer height of 0.1 mm), as well as under bending (up to 88.54 N for the same layer height), attributed to favourable fibre alignment and better load distribution. Conversely, higher angles (60° to 90°) drastically reduced strength (tensile failures due to delamination; bending forces as low as 30.02 N for a layer height of 0.3 mm, highlighting the weakness of perpendicular layer interfaces. Furthermore, lower layer height could result in better overall mechanical properties. In conclusion, FDM parameters with low print angles and reduced layer heights are essential for maximising the mechanical robustness and structural integrity of PLA-CF parts, enabling the identification of improved production processes for industrial applications and educational prototypes, among others.

## 1. Introduction

Three-dimensional printing has led to significant advances in manufacturing processes and engineering sciences. Among other processes, this technology is used in industry through a technique known as Fused Deposition Modelling (FDM). This process facilitates the prototyping of mechanical parts or assemblies without the need to manufacture them in more expensive and difficult-to-manufacture materials. However, the development of this type of technology is limited due to the low mechanical resistance indices of the materials used in this process. Therefore, it is advisable to use materials with better mechanical properties and to determine the manufacturing process that will yield the best mechanical performance.

There is a variety of 3D printing polymers. However, the properties of an FDM-printed element are inferior due to the lines that separate the molten deposition between each layer of height, thus limiting the use of this method of manufacturing parts [1,2].

Over the last decade, new composite materials have been developed for 3D printing. In some of these, small or long fibres are added to a matrix in order to reinforce the materials and create products with greater resistance. Carbon fibres are a type of reinforcement that has become widely used. However, different parameters such as the orientation of the fibre within the polymer matrix and its length can significantly alter the properties [3,4].

Some studies have shown that incorporating short fibres into thermoplastic materials can reduce separation between printed layers and significantly improve mechanical performance [5]. Other studies have incorporated the addition of industrial waste and waste fibres into the raw material for manufacturing using 3D printing, finding improvements in the mechanical properties of the composites. Additionally, it has been indicated that the dominant fracture mechanism in these composites is fibre detachment [6,7].

With regard to the angle of inclination of printing on the test specimens, it is argued that their properties vary. According to [8], maximum tensile strength is affected by an increase in the angle. Therefore, the angle that generates the highest resistance is 0° due to the orientation of the load being parallel to the raster. Furthermore, it is concluded that a 45° angle generates more ductile behaviour. This means that in the manufacture of parts using the FDM method, the properties can be significantly high or low depending on their manufacture and additives, or fibres [9].

There is a wide variety of polymeric materials, which can be subdivided into two types: natural and synthetic polymers. Additionally, they can be classified according to the materials that comprise them, such as thermoplastics, elastomers, or thermosets. Therefore, the crystallinity of a polymer is of great importance because this characteristic defines physical properties such as melting point, hardness, modulus, tensile strength, and rigidity [10].

Polylactic acid (PLA) is a thermostable polymer derived from renewable sources such as corn and sugar cane, with mechanical properties similar to those of traditionally used engineering materials. However, it is a material that undergoes easy hydrolytic degradation and is easy to print due to its good adhesion to the heating bed and excellent interlayer adhesion [11]. It should be noted that the mechanical properties of PLA make it ideal for use in biomedical devices or for printing study models in cardiovascular healthcare [12].

Other applications of 3D printing are in the field of education, where it has been demonstrated that its use can influence the cognitive processes involved in learning content [13,14].

Among the materials used in 3D printing, Carbon Fibre-reinforced Polylactic Acid (PLA-CF) is a polymer with better mechanical properties than common PLA [15], Some research carried out on this filament, varying parameters such as the pattern and fill densities as well as table orientation, concludes that the fill density is directly proportional to the strength of the test pieces, also due to the qualities of the material [16].

The demand for new materials with improved properties is constant, and research has been conducted into composite filaments consisting of mixing fibres with a polymer matrix. This is reflected in various characteristics such as increased thermal conductivity, lower thermal expansion, providing greater dimensional accuracy, and a reduction in residual stresses [17].

For the development of this research, the use of technical standards that standardise tests to characterise test specimens according to the physical property of interest is of great importance in order to establish a standard with which other researchers can also carry out the same study and compare results. The standard for tensile testing of plastic test specimens with carbon fibre reinforcement is ASTM D3039 [18], which standardises a uniaxial tensile speed of 5 mm/min [19].

The results of tensile tests on carbon fibre-reinforced polymer specimens are affected by printing parameters, as stated by the researcher [20], printing speed and temperature can affect the bond interface between the polymer matrix and the fibre reinforcement. In addition to this, the vertical angle (0°) causes the test specimens to break more easily, overcoming the adhesive forces that bind the printed layers with less stress [21].

In the case of bending tests, the ASTM D790 [22] technical standard is used to determine the maximum bending load of a test piece supported at two points. For the characterisation of carbon fibre in bending tests, it was found that with a 5% reinforcement of fibres in the polymer matrix, the bending properties can be improved by approximately 11.82%. This demonstrates that there is an improvement in its properties [23].

Various researchers have concluded that the angle of impression, depending on its variability, is directly proportional to its properties [24]. The results obtained from various studies focus on the fact that the influence of tensile strength depends fundamentally on the filling density due to the greater mass present in the test piece [25]. Therefore, the layer width has a significant influence because thicker layers generate fewer void lines, resulting in better mechanical properties [26].

Researchers have studied the mechanical behaviour of PLA and carbon fibre-reinforced PLA (PLA-CF) with different orientations and thicknesses, finding that properties vary depending on thickness and printing direction. Carbon fibre reinforcement improved strength in the X and Y orientations but reduced it in the Z orientation due to low interlayer adhesion, indicating the need to improve parameters for structural applications [27].

In the research carried out by [28], the incorporation of carbon fibre into PLA (PLA-CF) was analysed to determine how it modifies the mechanical properties of FDM-printed parts. Samples with different proportions of PLA/PLA-CF (6–27%) were manufactured, and it was found that a higher PLA-CF content increases tensile strength but reduces flexural strength, revealing a trade-off between stiffness and flexibility. Intermediate ratios (40–60% PLA-CF) achieved the best balance.

However, no evidence was found in the literature reviewed of research showing how the print angle and layer height affect the tensile and flexural strength of PLA-CF. The development of this work involves a novel experimental design to determine the tensile and flexural strength of carbon PLA specimens using ASTM D3039 [18] and ASTM D790 [22], respectively, varying the layer height (0.1 mm, 0.2 mm, and 0.3 mm) and orientation of the piece (0°, 15°, 30°, 45°, 60°, 75°, and 90°). The correlation of the results obtained with the aforementioned variables allows the optimal combination of parameters to be obtained. This demonstrates that it is possible to determine optimal production processes for use in start-ups and the development of prototypes used in education.

## 2. Materials and Methods

The Fused Deposition Modelling (FDM) process begins with the use of CAD software, Solidworks 2024, to design the solids to be manufactured. Once the design has been created, the most commonly used format is STL (Standard Tessellation Language), which represents the model as a mesh of triangles [29].

The STL file is then imported into a slicer programme, which converts the model into a series of successive layers distributed along the *Z*-axis. This programme generates G-code that the printer interprets to manufacture the part in the printer.

The printer consists of a bed or base that facilitates the adhesion of the material during printing. In addition, the filament, which can have a diameter of 1.75 mm (the most common) or 2.85 mm (less common), is fed into the extrusion system.

The extruder consists of a stepper motor and a pair of gears that pull the filament and send it to the hotend. The hotend, which is responsible for melting the filament and depositing it on the base, consists of an aluminium cube with an internal heating element that raises the temperature of the material to over 200 °C, reaching its melting point. This allows the filament to flow through the nozzle and be deposited layer by layer, thus forming the final piece.

### 2.1. Print Settings

In the programmes used for the FDM process, it is possible to modify printing parameters such as layer height, fill density, fill pattern, temperatures, printing angle, supports, etc. These parameters make it possible to manufacture elements according to the required needs. These are therefore the fundamental parameters for starting in 3D printing.

Layer height refers to the thickness of a layer of deposited material. It is defined as the vertical displacement applied by the *Z*-axis motor to create thin or thick layers. When printing layer heights starting from 0.1 mm, mechanical parts are obtained with a better-looking surface finish and a smooth appearance because the layers are less visible. Unlike a higher layer height, the print lines will be more noticeable.

Fill density is defined as the percentage of fill of the entire internal volume of a part [30]. It is possible to print with percentages ranging from 0 to 100%. Increasing the fill density to the maximum option implies high production costs due to the use of greater quantities of material and longer printing times.

The fill pattern refers to the type of internal geometry of the volume of a printed part. There is a wide variety, the most common being linear, triangular, hexagonal, centroid, or concentrated [31]. Temperature is the physical magnitude of thermal energy generated from a resistor located in the printer’s hotend. The purpose of applying temperature to the polymeric material is to bring it to a melting temperature (Tm) and generate adhesion to the print bed [32].

The print angle refers directly to the inclination measured in degrees (°) relative to the print bed and represents the direction in which an element will be printed. The inclination can be configured in the slicer used in additive manufacturing. Depending on this angle, the mechanical strength of a part can increase or decrease depending on the load applied [33].

Supports refer to the creation of auxiliary structures that serve to hold up parts of a piece that would otherwise be suspended in the air during manufacturing and could not be printed, as the material would simply fall and form knots. They are generally used on overhangs exceeding 45°, or on elements that lack a solid base and cannot guarantee their stability from the first layer [34].

The material used for this study is Carbon Fibre-reinforced Polylactic Acid (PLA-CF) and the printing parameters are presented in Table 1.

The samples were printed using a Creality CR-10 Smart Pro printer (Creality Co, Shenzhen, China). Samples for tensile testing are prepared according to ASTM D3039, and samples for three-point bending test are prepared according to ASTM D790.

An Armotec EXCT-PW910LCD (United Scope, Irvine, CA, USA) digital stereoscope was used to obtain fractographic images of the fault surfaces.

**Figure 1:**
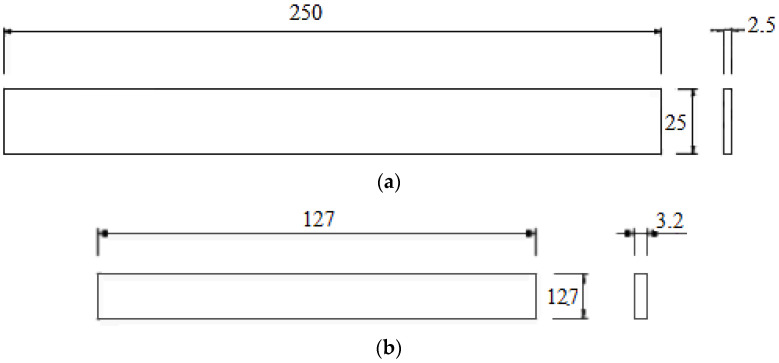
(**a**) ASTM D3039 tensile sample dimensions (mm); (**b**) ASTM D790 three-point bending sample dimensions (mm).

Figure 1a shows the dimensions of the tensile samples used, while Figure 1b shows the dimensions of the three-point bending samples used.

The printing specifications are shown in Table 2.

### 2.2. Experimental Design

The proposed experimental design is based on the need to quantify the impact of critical FDM printing parameters—printing angle (θ) and layer height (h)—on the mechanical properties of carbon fibre-reinforced PLA (PLA-CF). The selection of a factorial model with replicates (n = 5) allows us to analyse not only the individual effects of θ and h, but also their interaction, which is critical in composite materials due to the anisotropy induced by the orientation of the fibres during extrusion [3]. This approach maximises statistical efficiency by covering representative combinations: angles of 0°, 15°, 30°, 45°, 60°, 75°, and 90° (parallel alignment, in multiple diagonals and perpendicular to the load), and layer heights of 0.1 mm, 0.2 mm, and 0.3 mm (FDM printing ranges that affect interlaminar consolidation).

The total number of specimens used during this research is 210, and the details are given in Table 3.

**Figure 2:**
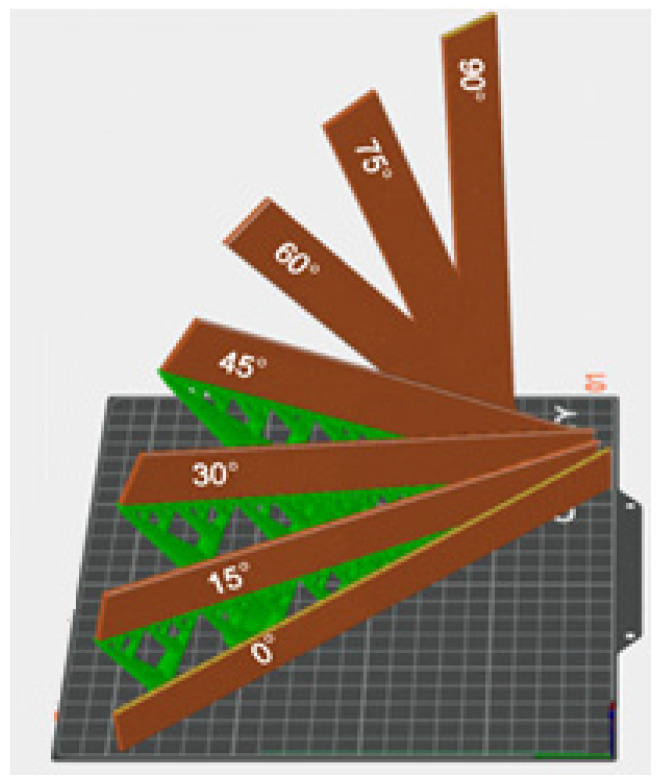
Angles of the impression samples.

Figure 2 provides an overview of the printing status of the test specimens.

**Figure 3:**
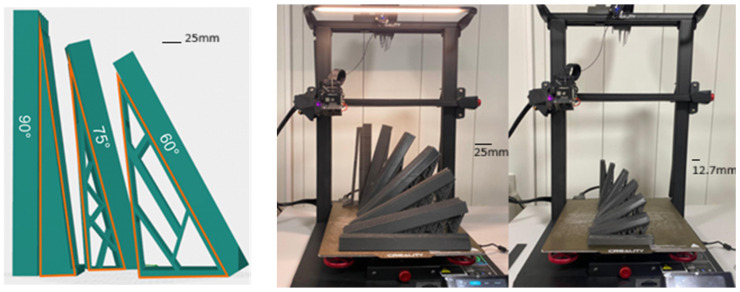
Support structures of 3D printing specimens.

In order to guarantee the print quality of the samples, taking into account the problem of the suspension angle during the printing process, support structures are pre-established in the printing software (Orca Slicer v2.3). The support structures (SS) are illustrated in Figure 3.

### 2.3. Uniaxial Tensile Experiment

Uniaxial tensile tests are performed on the Haida HD-B604-S (Haida, Guangdong, China) computer-controlled electronic universal tensile testing machine with a capacity of 20 kN. In all tensile tests, ASTM D3039, the samples are stretched at a constant speed of 2 mm/min and at an ambient temperature maintained at 23 °C. Therefore, the test conditions are standard.

Prior to testing, the actual width of each specimen will be verified by measuring at three different points, discarding those with variations >5% from the nominal value (25 mm). Force and displacement data will be acquired at 100 Hz to eliminate vibrations

The five replicates per condition will allow the complete factorial experimental design to be applied, ensuring a 95% confidence interval in the results.

### 2.4. Three-Point Bending Test

For the development of three-point bending tests, a Haida HD-B604-S universal testing machine with a capacity of 20 kN and a three-point bending accessory is used, where the base will have two points on which the test piece will rest and the other loading point on which the force is generated on the test piece, in accordance with ASTM D790, using a load head with a semi-spherical tip with a radius of 5 mm. The load application speed will be set to 2 mm/s, corresponding to the standard method for rigid plastic materials.

The force and displacement data will be acquired at 100 Hz to eliminate vibrations.

### 2.5. Statistical Analysis

All the data obtained in the evaluations were subjected to analysis of variance at a significance level of *p* < 0.05; if statistically significant differences were found, Tukey’s multiple comparison test was employed. All analyses were performed using the free version of statistical software InfosStat V2020.

## 3. Results

### 3.1. Uniaxial Tensile Tests

**Figure 4:**
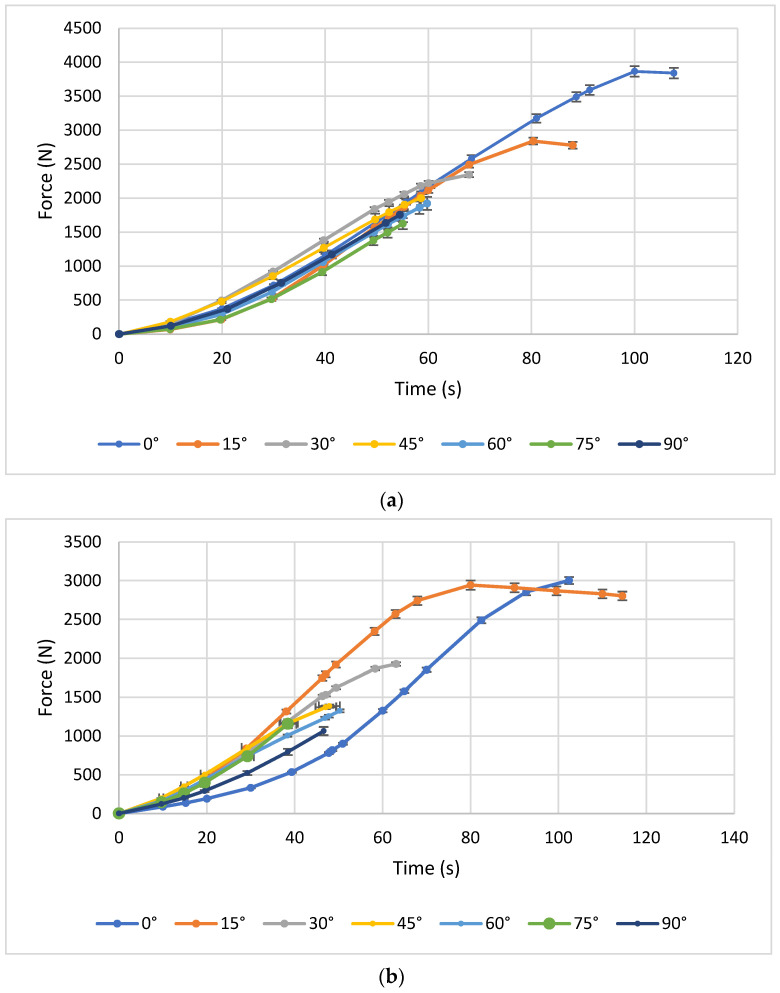

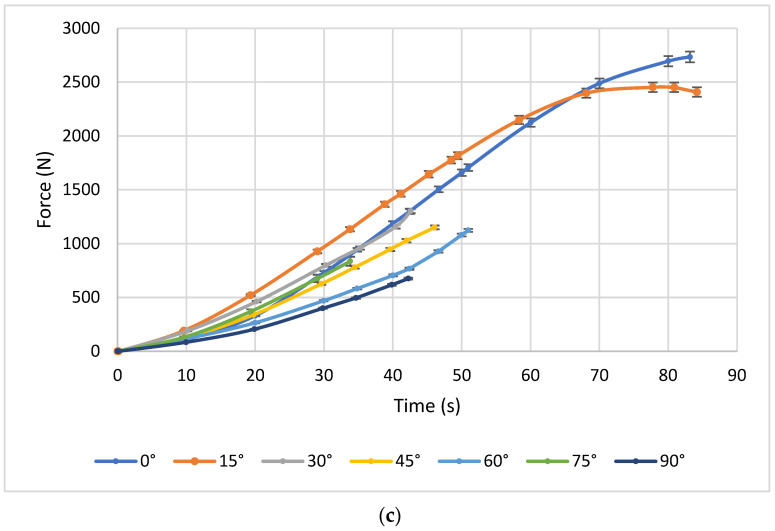
Tensile curves for PLA–carbon fibre (PLA-CF) composites printed with three different Layer heights: (**a**) 0.1 mm, (**b**) 0.2 mm, and (**c**) 0.3 mm.

Figure 4 illustrates representative maximum force curves for PLA–carbon fibre (PLA-CF) composites printed with three different layer heights: 0.1 mm, 0.2 mm, and 0.3 mm. Each subgraph corresponds to a different printing angle and shows how the mechanical behaviour evolves as the angle increases (0°, 15°, 30°, 45°, 60°, 75°, and 90°).

**Figure 5:**
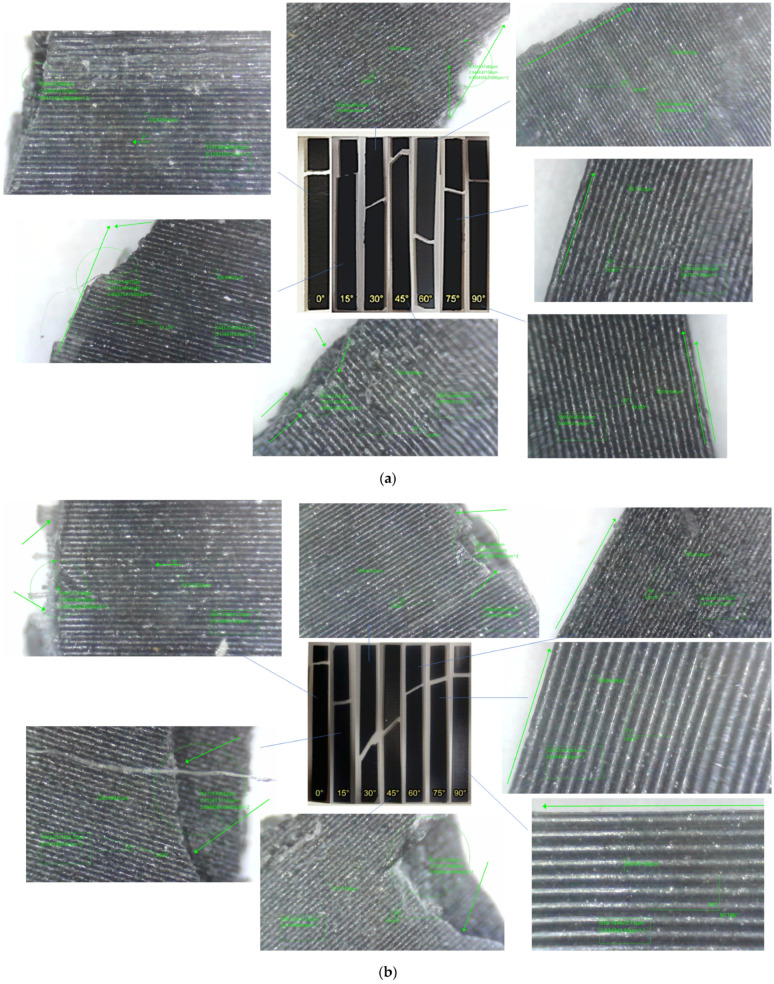

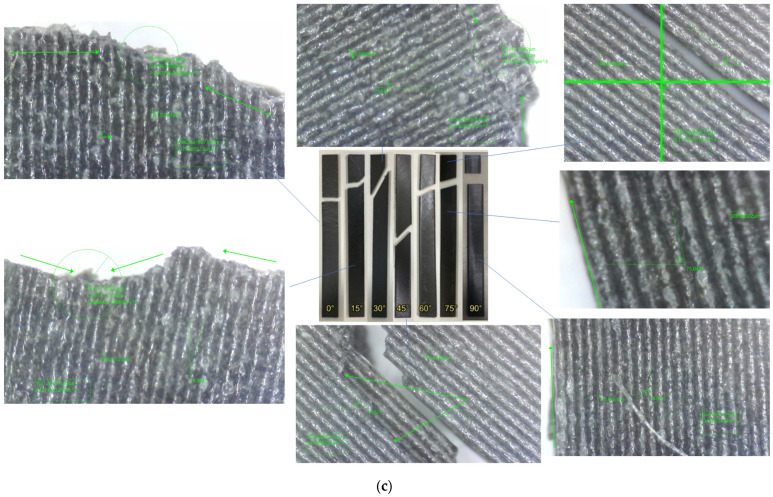
Failure features and details of specimens in Uniaxial tensile test with three different layer heights: (**a**) 0.1 mm, (**b**) 0.2 mm, and (**c**) 0.3 mm.

Figure 5a shows the tensile behaviour of printed specimens with a constant layer height of 0.1 mm and different printing angles. A significant variation in the maximum load supported can be observed. The specimen printed at 0° shows the highest behaviour, reaching values close to 4000 Newtons. This is followed by the test piece printed at 15°, with an average behaviour ranging between 2500 and 3000 Newtons. Finally, the test pieces printed at 30°, 45°, 60°, 75°, and 90° show lower performance, with maximum loads of less than 2500 N, indicating that these orientations are less favourable for tensile strength when using a layer height of 0.1 mm.

When analysing the results with a layer height of 0.2 mm and different printing angles, in Figure 5b, two similar behaviours can be distinguished between the angles of 0° and 15°. The remaining configurations (30°, 45°, 60°, 75°, and 90°) continue to show lower strength compared to the 0° and 15° orientations for the 0.2 mm layer height.

In Figure 5c, for a layer height of 0.3 mm, similar behaviour is identified at angles of 0° and 15°, with load magnitudes close to 2500 N. In contrast, angles of 30°, 45°, 60°, 75°, and 90° show the worst results, with loads ranging from 1500 to 2000 N, making them very unfavourable in terms of mechanical strength for the manufacturing conditions evaluated.

Figure 5 shows a comparison of the failure patterns obtained in the tensile tests for specimens with different impression angles (0°, 15°, 30°, 45°, 60°, 75°, and 90°). Variations in the fracture line can be identified depending on the impression angle, demonstrating differences in the mechanical behaviour of the printed parts.

Table 4 shows the ANOVA statistical analysis applied to the tensile test, which has been used to determine the significant effect of factors on the established parameters. The results indicate, with a confidence level of 95%, that both the impression angle and the layer height have a statistically significant influence on the maximum tensile strength of carbon fibre-reinforced PLA material. This result is based on the *p*-values obtained, all of which are less than 0.05.

When the layer height is kept constant at 0.1 mm, the 0° printing angle generates the highest average force value, with a significant statistical difference compared to the lowest value obtained at 90° and 0.3 mm of layer height. This difference is explained by the orientation of the layers: at 0°, the fibres are completely aligned with the load, facilitating efficient stress transfer and minimising interlaminar failure, whereas at a 90° printing angle, it supports less load. These results are according to those found by [36], who agree on the increase in load related to the variation in the angle of printing and the transition in the various failure modes that depend on the angle.

**Figure 6:**
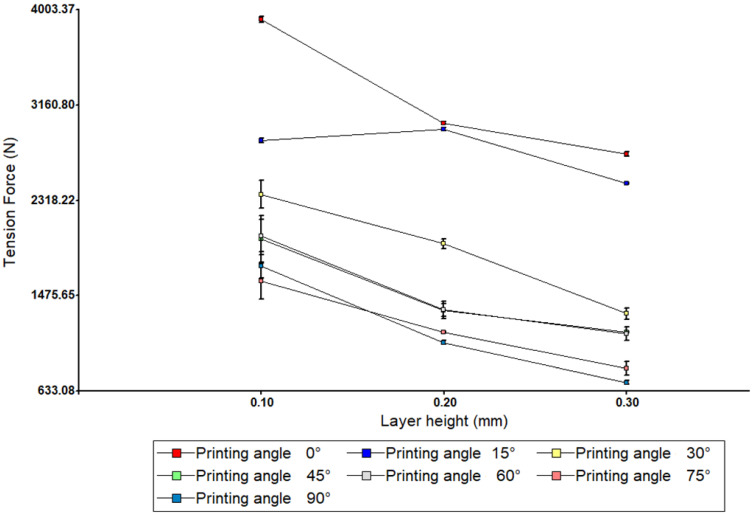
Two-way analysis of variance (ANOVA). Tensile testing.

In order to identify the interaction between the angle of impression and the height of the layer, Figure 6 shows a two-way ANOVA graph.

It can be observed that for most angles between 0° and 15°, the tensile force remains stable or increases slightly with increasing height. The figure shows that the lines on the graph are not parallel, and some intersect, indicating a significant interaction. The non-parallelism of the lines in the interaction graph suggests that the effect of layer height on mechanical strength depends on the printing angle. This interaction means that it is not possible to optimise each parameter separately. For example, while a layer height of 0.10 mm improves strength at angles such as 0°, it does not have the same effect at angles such as 90°. From a statistical point of view, the interaction term in the ANOVA (Angle × Height) has a *p*-value < 0.05, confirming its significance.

### 3.2. Three-Point Bending Tests

**Figure 7:**
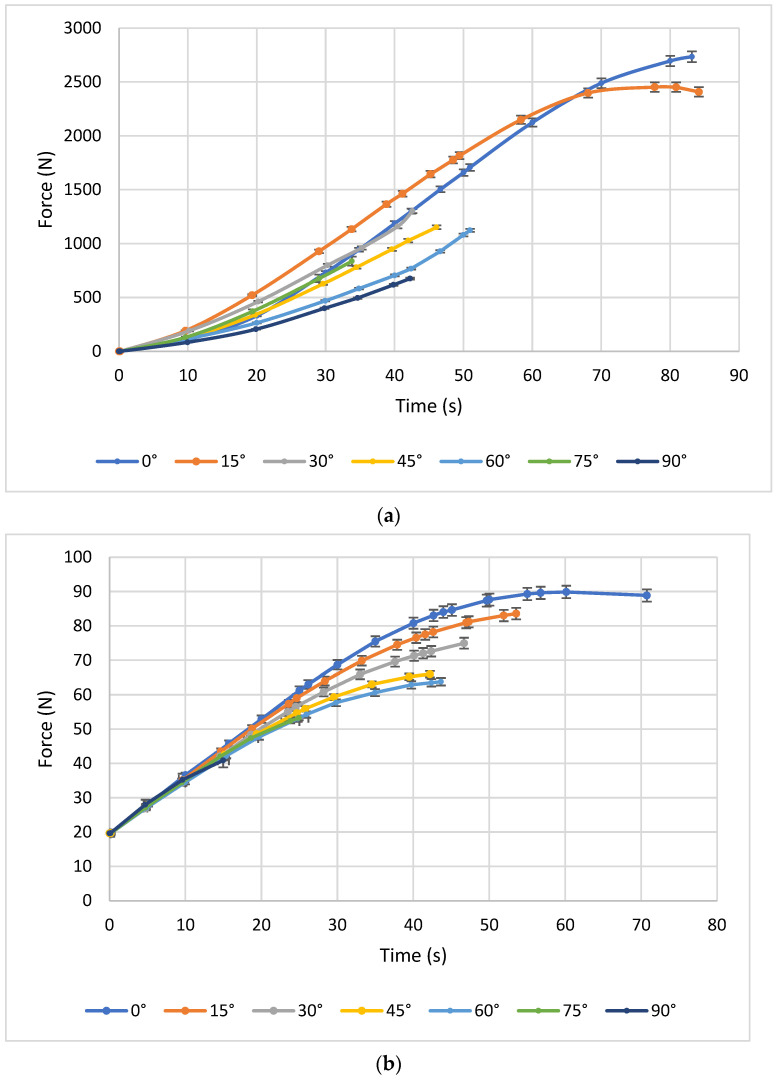

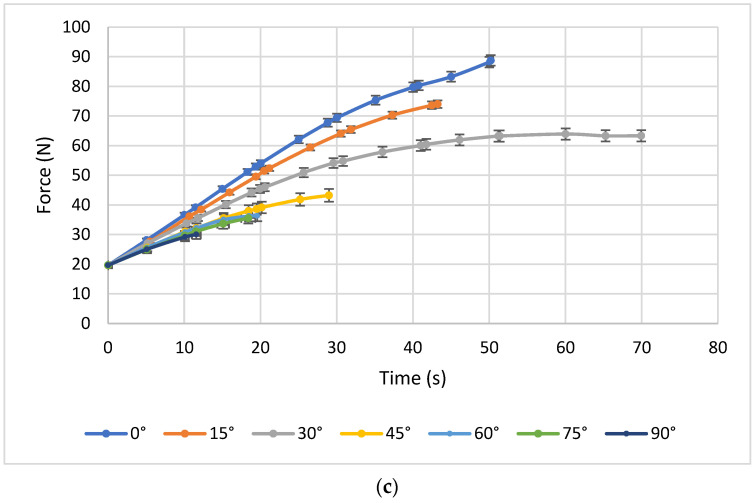
Bending curves for PLA–carbon fibre (PLA-CF) composites printed with three different Layer heights: (**a**) 0.1 mm, (**b**) 0.2 mm, and (**c**) 0.3 mm.

Figure 7 illustrates representative maximum force curves for PLA–carbon fibre (PLA-CF) bending test, composites printed with three different layer heights: 0.1 mm, 0.2 mm, and 0.3 mm. Each subgraph corresponds to a different printing angle and shows how the mechanical behaviour evolves as the angle increases (0°, 15°, 30°, 45°, 60°, 75°, and 90°).

In Figure 7a, for a constant layer height of 0.1 mm, it can be seen that the printing angles of 0°, 15°, and 30° exhibit relatively homogeneous behaviour in terms of flexural strength, indicating a similar influence of these orientations with this layer height. The test piece printed at 45° shows average strength, approaching 80 Newton. Meanwhile, the angles of 60°, 75°, and 90° exhibit low flexural strength compared to the smaller angles. This suggests that when layers are deposited at larger angles to the direction of the force applied in flexure, the strength of the test piece decreases significantly.

Figure 7b shows the results of the bending test with a layer thickness of 0.2 mm and all print angles. For a layer height of 0.2 mm, the trend remains the same. The 0° and 15° angles show superior flexural strength, with magnitudes above 80 Newton, indicating better mechanical behaviour compared to the other angles for this layer height. This result suggests that, for a slightly higher layer height, orientations close to alignment with the bending stress remain the most favourable.

Figure 7c shows the results of the bending test with a layer thickness of 0.3 mm and all print angles. In this graph, with a layer height of 0.3 mm, lower flexural strength is observed compared to the 0.2 mm graph for all angles. This supports the idea that a greater layer height can have a negative influence on mechanical properties under bending, regardless of the printing angle. Adhesion between layers could be less effective with a greater layer height, which would affect the overall resistance of the test piece under bending.

Table 5 shows an analysis of variance (ANOVA) applied to the bending tests, which has been used to determine the significant effect of factors on the established parameters. The results indicate that both the printing angle and the layer thickness have a statistically significant influence on the mechanical strength of carbon PLA.

For layer heights of 0.1 mm, 0.2 mm, and 0.3 mm, the results are based on the *p*-values obtained, all of which are less than 0.05. It is identified that with printing angles of 0°, the highest bending load values are obtained at all layer heights considered. This coincides with the results reported by [37], who indicate that the maximum bending load is found in specimens with lower layer heights and constant thicknesses.

**Figure 8:**
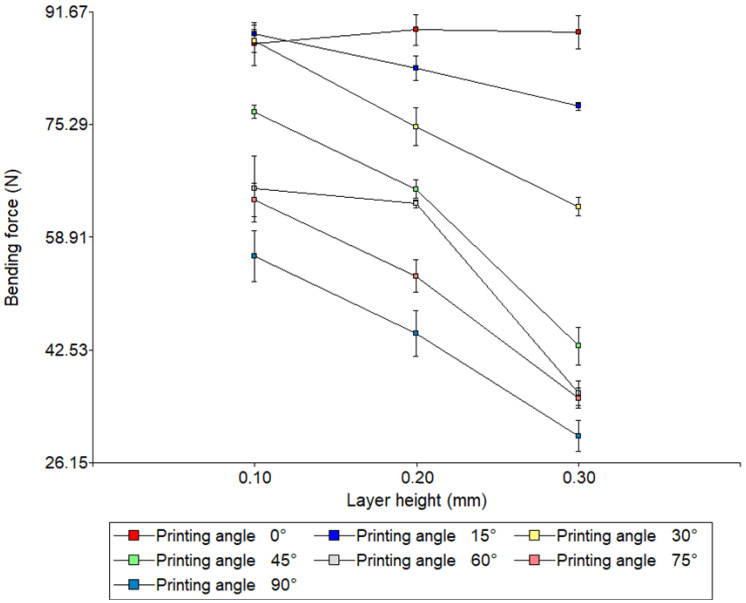
Two-way analysis of variance (ANOVA). Three-point bending test.

In order to identify the interaction between the angle of impression and the height of the layer, Figure 8 shows a two-way ANOVA graph.

Analysis of the interaction between layer height and print angle revealed significant effects on the flexural strength of 3D-printed specimens. In general, flexural strength was observed to decrease as layer height increased, indicating that thinner layers (0.10 mm) favour greater mechanical integrity. On the other hand, the printing angle showed a marked effect: specimens printed at low angles (0–15°) achieved the highest flexural strengths, while those manufactured at high angles (75–90°) had considerably lower values.

The interaction between the two factors was evident in the ANOVA graph, as the lines corresponding to the different angles were not parallel. This suggests that the effect of layer height depends on the printing angle. For example, at 90°, the difference between 0.10 mm and 0.30 mm was pronounced, while at 0°, the variation between heights was minimal. This confirms that the printing configuration critically influences the mechanical properties of parts manufactured using additive manufacturing. In particular, the combination of thin layers and low angles is the most effective strategy for maximising flexural strength, while configurations with thick layers and high angles should be avoided in applications requiring high structural integrity.

## 4. Discussion

### 4.1. Analysis of Uniaxial Tensile Tests

Based on the results obtained in the tensile test, it can be observed that both the layer height and the print angle have a significant impact on the tensile strength of carbon PLA specimens manufactured according to ASTM D3039. In general, print angles close to 0° and 15° tend to offer greater strength, while larger angles, especially 90°, result in lower load capacity. The influence of layer height varies depending on the print angle considered; however, for layer thicknesses of 0.1 mm, the highest load magnitudes are obtained.

**Figure 9:**
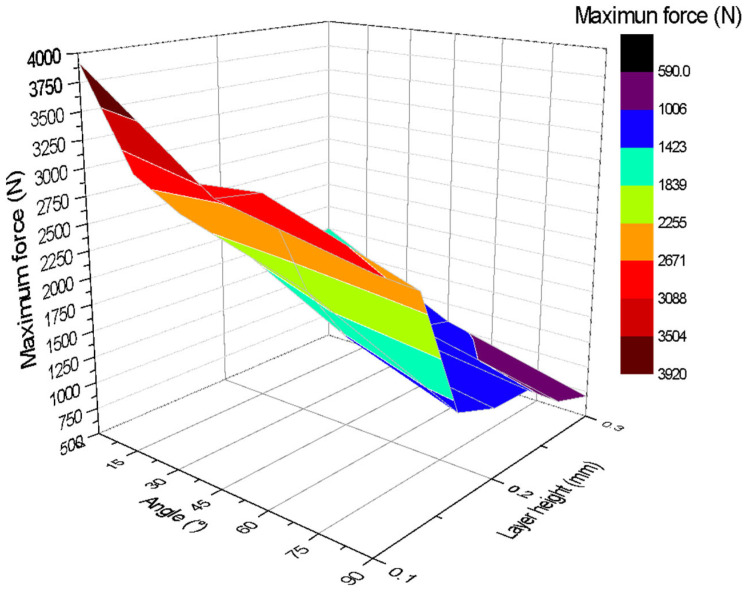
Three-dimensional graph for the Uniaxial tensile tests.

Figure 9 shows a three-dimensional graph illustrating the relationship between the angle of impression (*X*-axis), layer height (*Z*-axis), and maximum force applied (*Y*-axis). The colours on the graph indicate the magnitude of the maximum force, showing that the best mechanical strengths are obtained with printing angles close to or equal to 0° and a layer height of 0.1 mm. The highest value recorded in the test, close to 3920 N, is found in this region of the graph. Similarly, the results with the lowest mechanical strength are obtained in specimens printed at 90° with a layer height of 0.3 mm.

The tension diagram confirms that the highest force magnitudes correspond to test specimens with layer heights close to 0.1 mm and inclination angles of 0°. This is explained by the fact that with this orientation, the fibres or directions of the layers are perpendicular to the axial load, and the layers have the greatest possible length within the test specimen.

**Figure 10:**
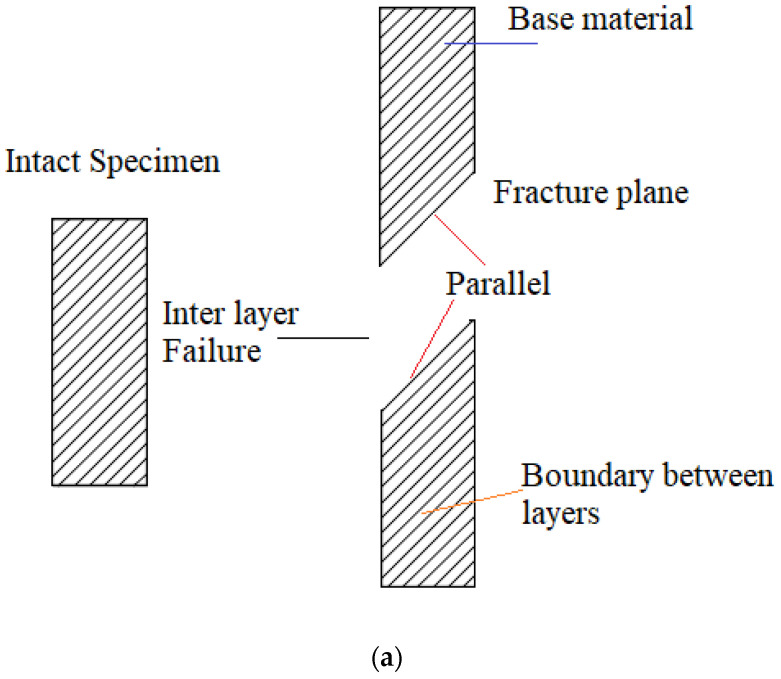

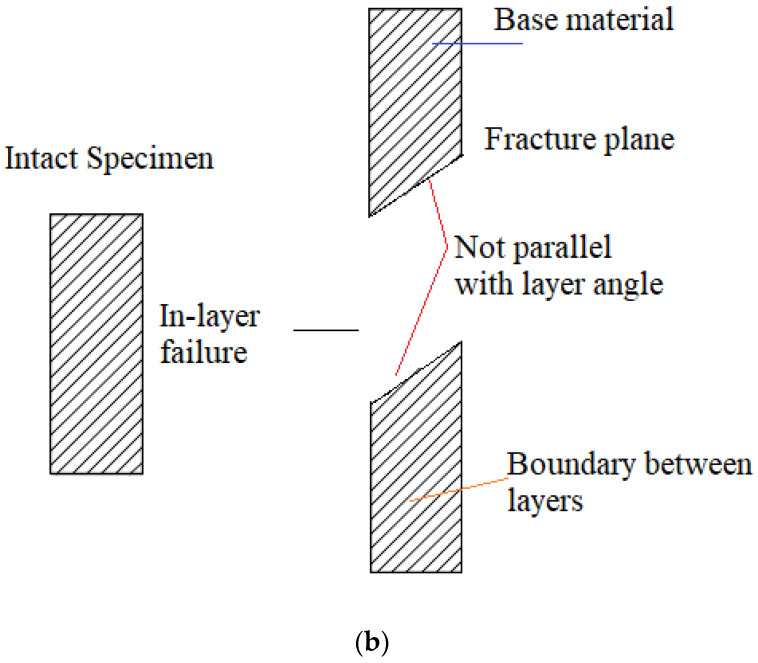
Failure mode: (**a**) failure mode between layers and (**b**) in-layer failure mode.

In order to analyse the experimental results, it is necessary to define the two failure modes: interlayer failure mode and in-layer failure mode, which are illustrated in Figure 10. The interlayer failure mode occurs when the fracture appears at the interface between two adjacent layers of material, and the layers of material remain intact after failure; the failure surface is parallel between each of the layers of the fracture plane.

In-layer failure mode occurs when the layers of material break, and the failure surfaces and broken layers of material are not parallel to each other [26,38].

**Figure 11:**
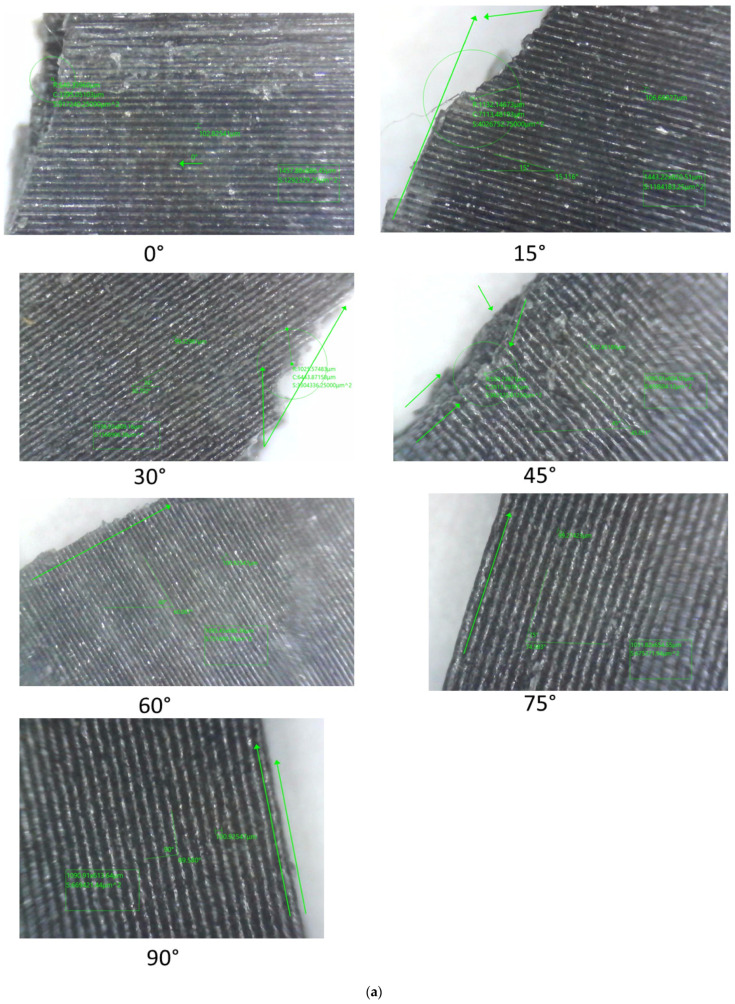

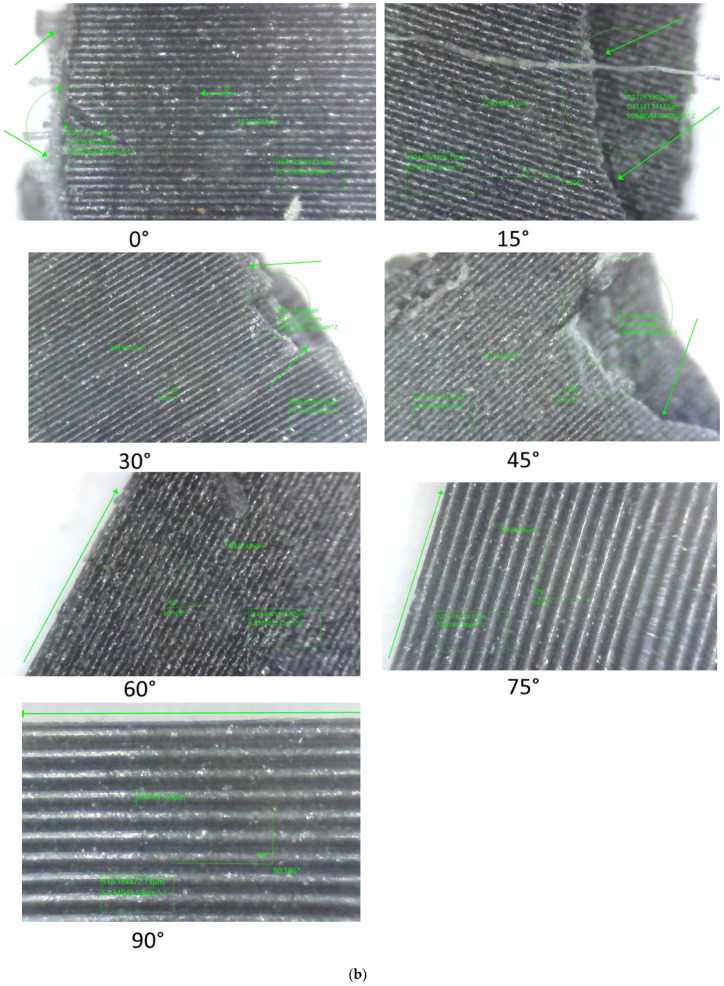

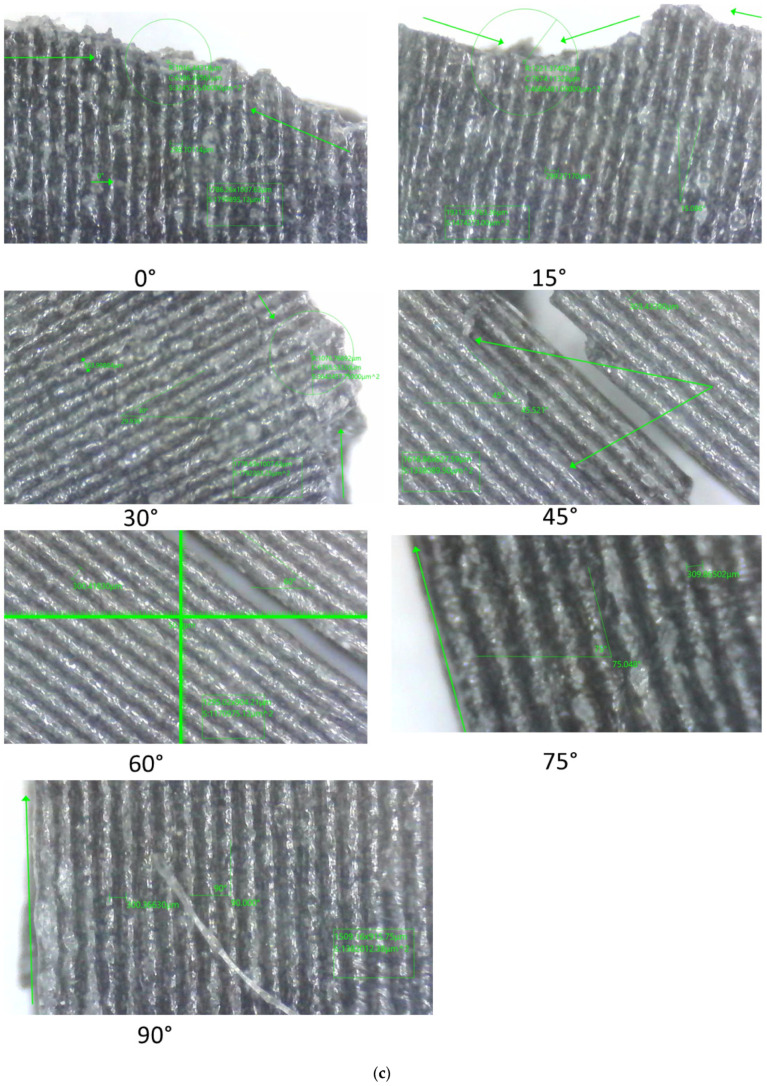
Failure details of samples in Uniaxial tensile test with three different layer heights: (**a**) 0.1 mm, (**b**) 0.2 mm, and (**c**) 0.3 mm.

The results of the experimental tests for the layer heights under study are shown in Figure 11. The failure characteristics are shown enlarged.

Analysis of the fracture interface using optical microscopy revealed that specimens with angles between 45° and 90° showed poor interfacial bonding, coinciding with a weak interfacial bond between the layers and the matrix, particularly with greater layer heights, causing them to separate. This coincides with the observations made in the research carried out by [19].

Therefore, the interlayer failure mode and the in-layer failure mode can be seen, it can be observed that for specimens printed at 0°, the loads are aligned with respect to the print, in addition to a cut in the test piece that is completely perpendicular to the tensile load. This result confirms that the 0° angle maximises tensile strength because the layers are aligned with the force, which secures and minimises weakness between layers. Another factor to note is the presence of white cracks near the cut area, demonstrating the entire stress zone to which the test piece was subjected.

Table 6 shows the classification of the faults observed. It can be seen that for specimens with angles of 60°, 75°, and 90°, inclined fractures parallel to the layer direction are observed, located in the material matrix, which leads to failures due to fractures at the interface between layers. This is indicative of inter-layer failure mode and is associated with the fact that the specimens suffered fractures due to weak cohesion between the layers, demonstrating lower strength of the specimens manufactured with these orientations. In the case of specimens manufactured with orientations of 0°, 15°, 30°, and 45°, the direction of failure and the layer orientation angle do not coincide, which is indicative of an in-layer failure mode where the load is mainly supported by the material and not by the bonds between layers. As a result, the failure loads are higher than those observed at manufacturing angles greater than or close to the perpendicular to the failure load.

### 4.2. Analysis of Three-Point Bending Tests

The results of the bending tests reveal a clear influence of both the print angle and layer height on the flexural strength of the carbon PLA test specimens. It is observed that print angles close to 0° and 15° generally provide the highest flexural strength, especially with layer heights of 0.1 mm and 0.2 mm. This is because the layers of material are more aligned with the direction of the bending stress, allowing for better load distribution across the layers.

**Figure 12:**
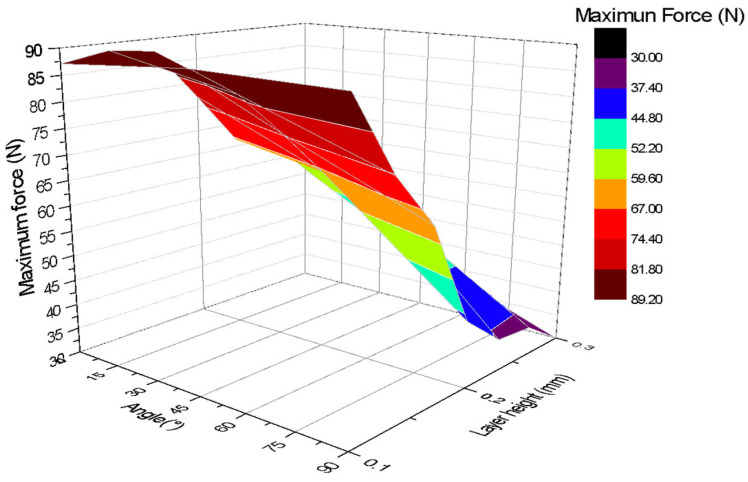
Three-dimensional graph for the Three-point bending tests.

Figure 12 shows a three-dimensional graph illustrating the relationship between the angle of impression (*X*-axis), layer height (*Z*-axis), and maximum force applied (*Y*-axis). The colours on the graph indicate the magnitude of the maximum force, showing that the best mechanical strength is obtained with print angles close to or equal to 0° and a layer height between 0.1 mm and 0.2 mm. The highest value recorded in the test, close to 90 N, is found in this region of the graph. It can be observed that the closer the angle of inclination is to 0°, the greater the force that the test piece can withstand.

The tension diagram indicates that specimens with a thickness of 0.1 mm generally withstand the maximum force, while those with thicknesses close to 0.2 and 0.3 mm tend to withstand less force. It also suggests that the highest forces occur for layer thicknesses close to 0.1 and 0.2 mm, because the fibres or layer directions are perpendicular to the load, and in this orientation the layers have the greatest length of the test piece. On the other hand, layer orientations that are parallel to the load were more prone to fracture.

As the print angle increases, flexural strength tends to decrease, with angles of 60°, 75°, and 90° exhibiting the lowest strength. In these orientations, flexural forces act more perpendicularly to the interlayer bond, which can promote delamination and premature failure.

Layer height also plays an important role. In general, it has been observed that a lower layer height (0.1 mm and 0.2 mm) tends to favour greater bending strength, especially at smaller print angles. This could be due to better adhesion between thinner layers. However, for the 0° angle, the differences in maximum strength appear to be less pronounced between the different layer heights, although ductility is affected.

The 45° angle represents a turning point where flexural strength begins to decrease significantly at all layer heights. These findings are important for improving the 3D printing parameters of parts that will be subjected to bending stresses, indicating that low printing angles and appropriate layer heights should be prioritised to maximise mechanical strength. Specimens with lower layer heights tend to deform more before breaking.

Various studies have been conducted on the mechanical behaviour of chopped carbon fibres. In the study carried out by [39], the mechanical properties and fracture toughness were studied experimentally and by finite element analysis (FEA). An inverse correlation was observed between tensile strength and fracture toughness, which indicates the importance of selecting the orientation to meet specific requirements. This element has been extensively studied in the research carried out, constituting a notable novelty.

In the research carried out by [19], four groups of specimens were prepared using FDM technology: pure PLA, PLA with short carbon fibre (PLA-SCF), PLA printed with continuous carbon fibre (PLA-CCF), and PLA-SCF printed with CCF (PLA-SCF-CCF), with the aim of studying the mechanical performance and comprehensive response of the tensile and flexural properties. Printed in a flat unidirectional configuration of 0°. The results showed that PLA-SCF exhibited a tensile strength of 43.75 MPa, almost identical to that of pure PLA (43.83 MPa), up to 460% lower than the strength obtained in PLA-CCF.

In comparison, the novelty of the developed article lies in seeking to improve the performance of short fibre composites (PLA-SCF), a material that often exhibits inadequate or limited mechanical properties in additive manufacturing. By identifying the print angle and layer height parameters, it is possible to maximise the structural robustness and integrity of PLA-CF parts.

## 5. Conclusions

The test results obtained in the mechanical tests allow for a critical analysis of the influence of printing parameters on the strength of PLA-CF. In particular, the variation in the printing angle had a dominant effect in both the tensile tests and the bending tests.

For the bending load, it was confirmed that configurations with low printing angles (0° and 15°) generate more favourable mechanical behaviour, reaching average maximum load values of up to 88.54 N, especially when a layer height of 0.1 mm is used.

This condition of direct alignment between the load direction and the orientation of the layers promotes efficient stress transfer and reduces the probability of delamination. As the impression angle increases towards 90°, the strength decreases dramatically, with average values as low as 30.02 N, which is attributed to the perpendicular action of the load on the interfaces between layers, where cohesion is structurally weaker.

In relation to layer thickness, it was observed that a lower height significantly improves mechanical strength. This trend is repeated in almost all of the printing angles analysed and is justified by the higher density of the deposition lines and the better adhesion between layers that occurs when thinner layers are used. ANOVA statistical analysis confirms that the differences observed are not random, but a direct effect of the printing factors, with a *p* < 0.05 for most combinations.

In contrast, samples manufactured with high angles (60–90°) generally exhibited lower tensile strengths, which is directly related to interlayer failure modes. This observation reinforces the need to maintain controlled and consistent layer orientations when seeking uniform structural properties in printed parts, with the best results occurring for low print angles and small layer heights.

Based on the identified failure modes, it is concluded that inter-layer failures tend to occur when the print angle is small, and in-layer failure modes tend to occur when the print angle is large.

## Figures and Tables

**Table 1:** Material properties of 3D printing polymers used PLA-CF.

Property	PLA Carbon (PLA-CF)
Base Material	PLA with chopped carbon fibre
Colour	Black
% Deformation at failure	8% (estimated)
Carbon Fibre Content	~15–20 wt%
Density (typical)	~1.24 g/cm^3^
Carbon Fibre Length	~100–150 μm (chopped)

From PLA filament for 3D printing [35].

**Table 2:** General printing settings.

Parameter	Description
Layer height	0.1 mm, 0.2 mm, 0.3 mm
Fill density	100%
Fill pattern	Lineal
Print temperature (used)	220° C
Bed temperature (used)	60 °C
Filament diameter	1.75 mm
Print speed	40 mm/s
Print sequence	5 units
Flow	100%
Layer ventilation	50%
Material Fluid %	95%

**Table 3:** The number of specimens used during this research.

Printing Angle (°)						
0°	15°	30°	45°	60°	75°	90°
Layer height = 0.1 mm						
Uniaxial tensile tests						
5	5	5	5	5	5	5
Three-point bending test						
5	5	5	5	5	5	5
Layer height = 0.2 mm						
Uniaxial tensile tests						
5	5	5	5	5	5	5
Three-point bending test						
5	5	5	5	5	5	5
Layer height = 0.3 mm						
Uniaxial tensile tests						
5	5	5	5	5	5	5
Three-point bending test						
5	5	5	5	5	5	5

**Table 4:** Test data obtained in the Uniaxial tensile tests.

		Tensile Test			
Layer Height	Printing Angle (°)	Tensile Strength (Mpa)	Average Value (N)	Stand. Desv.	CV (%)
0.1	0°	62.68	3917.49 ^L^	24.23	0.62
	15°	45.58	2848.74 ^J^	20.22	0.71
	30°	37.92	2370.13 ^H^	123.05	5.19
	45°	31.58	1973.9 ^G^	207.51	10.51
	60°	32.09	2005.38 ^G^	143.56	7.16
	75°	25.69	1605.43 ^E^	163.78	10.20
	90°	27.74	1733.82 ^F^	105.28	6.07
0.2	0°	48.04	3002.72 ^K^	11.41	0.38
	15°	47.16	2947.39 ^J,K^	15.27	0.52
	30°	30.95	1934.36 ^G^	42.92	2.22
	45°	21.55	1346.98 ^D^	55.86	4.15
	60°	21.56	1347.74 ^D^	74.08	5.50
	75°	18.40	1150 ^C^	7.91	0.69
	90°	16.95	1059.34 ^C^	17.11	1.61
0.3	0°	43.63	2727.16 ^I^	18.38	0.67
	15°	39.46	2466.37 ^H^	6.07	0.25
	30°	21.04	1314.99 ^D^	47.68	3.63
	45°	18.40	1150 ^C^	7.91	0.69
	60°	18.24	1140.09 ^C^	58.78	5.16
	75°	13.32	832.49 ^B^	57.09	6.86
	90°	11.32	707.78 ^A^	17.26	2.44

Averages with different letters are significantly different (*p* ≤ 0.05).

**Table 5:** Test data obtained in the three-point bending tests.

		Three-Point Bending Tests			
Layer Height	Printing Angle (°)	Flexural Strength (Mpa)	Average Value (N)	Stand. Desv.	CV (%)
0.1	0°	120.43	87.01 ^H^	3.15	3.62
	15°	122.55	88.54 ^H^	1.24	1.40
	30°	121.04	87.45 ^H^	1.61	1.84
	45°	106.80	77.16 ^F^	0.93	1.21
	60°	91.30	65.96 ^E^	4.79	7.27
	75°	89.08	64.36 ^E^	2.45	3.81
	90°	77.79	56.2 ^D^	3.68	6.55
0.2	0°	123.27	89.06 ^H^	2.24	2.51
	15°	115.56	83.49 ^G^	1.74	2.08
	30°	103.81	75 ^F^	2.71	3.61
	45°	91.18	65.88 ^E^	1.34	2.03
	60°	88.29	63.79 ^E^	0.55	0.87
	75°	73.73	53.27 ^D^	2.35	4.41
	90°	62.12	44.88 ^C^	3.33	7.41
0.3	0°	122.77	88.7 ^H^	2.41	2.72
	15°	107.89	77.95 ^F^	0.54	0.70
	30°	87.72	63.38 ^E^	1.30	2.06
	45°	59.67	43.11 ^C^	2.76	6.40
	60°	50.22	36.28 ^B^	1.80	4.97
	75°	49.15	35.51 ^B^	1.47	4.15
	90°	41.55	30.02 ^A^	2.26	7.54

Averages with different letters are significantly different (*p* ≤ 0.05).

**Table 6:** Classification of observed failures.

Layer Height (mm)	Failure Modes	
	In-Layer Failure	Inter-Layer Failure
0.1	0° 15° 30° 45°	60° 75° 90°
0.2	0° 15° 30° 45°	60° 75° 90°
0.3	0° 15° 30° 45°	60° 75° 90°

## Data Availability

The original contributions presented in this study are included in the article. Further inquiries can be directed to the corresponding author.
